# Association of Maxillofacial Fractures With Demographic and Dental Factors: The Roles of Edentulism, Posterior Occlusion, Canines, and Lower Third Molar

**DOI:** 10.1111/edt.70069

**Published:** 2026-03-22

**Authors:** Canan Uzun, Ceyhun Uzun, Savas Ozarslanturk, Emrah Kagan Yasar

**Affiliations:** ^1^ Department of Dentomaxillofacial Radiology, Hamidiye Faculty of Dentistry Health Sciences University İstanbul Türkiye; ^2^ Department of Plastic and Reconstructive Surgery, Faculty of Medicine Kocaeli University Kocaeli Türkiye; ^3^ Department of Dentomaxillofacial Radiology, Gulhane Faculty of Dentistry Health Sciences University Ankara Türkiye

**Keywords:** fracture, lower third molar, mandible, maxillofacial trauma, risk factors

## Abstract

**Background/Aim:**

This retrospective, two‐center study aims to improve understanding of fracture pathogenesis in maxillofacial trauma by examining variations related to sex, age, dental status, posterior occlusion, and the presence of lower third molars (LTM) and lower canines.

**Material and Methods:**

A total of 466 patients with maxillofacial fractures were analyzed. Statistical methods included Pearson's chi‐square, Mann–Whitney *U*, Kruskal–Wallis, and binary regression analysis.

**Results:**

Fractures were more common in males. The maxilla and mandible were the most frequently affected bones, with mandibular fractures most prevalent in the symphysis/parasymphysis (19.1%) and angle regions (16.1%). Age was positively associated with coronoid and body fractures and negatively associated with angle and symphysis/parasymphysis fractures (*p* < 0.05). Total edentulism and the absence of canines significantly increased the risk of body fractures (*p* = 0.001). Posterior occlusion had a protective effect against body, angle, and symphysis/parasymphysis fractures but increased the risk of condylar fractures (*p* < 0.05). Presence of LTM increased the likelihood of angle fractures by 3.8 times (*p* < 0.001).

**Conclusions:**

These findings may provide valuable insights that support earlier and more accurate diagnosis, as well as timely and appropriate treatment in patients with maxillofacial trauma.

## 1 Introduction

Maxillofacial fractures resulting from various incidents in daily life, such as traffic accidents, falls, or sports injuries, can affect people of all ages in society [1, 2, 3]. However, some fractures may be overlooked during initial patient assessments in hospitals, which can lead to increased hospital stays and treatment costs [4]. Additionally, delayed treatment of maxillofacial trauma can make reconstructive procedures more difficult [5]. Therefore, it is crucial to carefully examine the maxillofacial region, which already has a complex anatomy, in trauma patients with timely, adequate, and appropriate imaging. For this purpose, some researchers have suggested some decision tools to guide clinicians in selecting imaging for maxillofacial injuries [6, 7].

Based on the importance of understanding the fracture pathogenesis and risk factors of maxillofacial bones in patient assessment, some researchers have focused on specific age groups [1, 2, 3, 8] or certain specific regional fractures [9] or have emphasized concomitant fractures [10, 11]. Among facial bones, mandibular fractures are significant due to their high incidence in trauma patients [12] and their potential to prolong hospital stays [3]. Studies have been published suggesting that some dental factors may be associated with mandibular fractures. For instance, researchers have proposed that the location of the fracture line in a traumatized mandible—whether it occurs at the mandibular angle or in the condylar/subcondylar region—may be influenced by the presence of lower third molar (LTM) teeth [9, 13, 14]. Additionally, a study in the literature suggests that the occlusion of the posterior teeth of the lower and upper jaws may influence the location of the fracture line through the effect of force transmission [2].

This study aimed to contribute to a better understanding of the pathogenesis of fractures in maxillofacial trauma. For this purpose, we investigated whether there were any differences between male and female patients, different age groups, patients with and without posterior occlusion, and patients with and without specific teeth such as LTMs and lower canines in patients who had fractures in the maxillofacial region. We also attempted to identify accompanying fractures. Our ultimate goal is to contribute to a better understanding of these aspects of maxillofacial fractures in order to focus more effectively on high‐risk areas, especially in cases of multiple fractures. This will help ensure that all fracture lines are not overlooked, particularly during early assessments.

We hypothesized that there would be differences in the number and localization of fractures between young and elderly patients, female and male patients, patients with and without posterior occlusion, patients with teeth and those who are totally edentulous, and patients with or without specific teeth such as LTMs and lower canine teeth.

## 2 Materials and Methods

The archives of the Health Sciences University, Faculty of Dentistry, Dentomaxillofacial Radiology Department and the Kocaeli University, Faculty of Medicine, Plastic and Reconstructive Surgery Department were screened retrospectively from the most recent to the oldest between 2016 and 2025. Cases meeting the specified criteria were included in the study.

Inclusion criteria

– Indication for cone beam computed tomography (CBCT) or computed tomography (CT) scan due to trauma or suspected fracture.
– Accessibility of images.
– The images are of sufficient diagnostic quality.
– The patient is 20 years of age or older.
– The patient has no cysts, tumors, fibroosseous lesions, metabolic lesions, or infectious lesions in the jaw and facial bones.

Exclusion criteria

– Fracture lines caused by non‐traumatic causes, such as firearm injuries.
– Previous injuries.
– Inability to access images.
– Images that are not of sufficient diagnostic quality.
– The patient is under the age of 20.
– Presence of cystic, tumorous, fibroosseous, metabolic, or infectious lesions in the maxillofacial region.

The sample size for the study was calculated based on the number of affected mandibles required, using the study by Kandel et al. [15] as a reference. G Power software version 3.1 [16] was used to calculate the sample size. A total of 237 fractured mandibles were needed to detect a significant difference using the “goodness of fit” test, with a type I error (*α*) of 0.05, a power (1‐β) of 0.88, and an effect size of 0.222. Archives were screened retrospectively from the most recent to the oldest until this number was reached. All patients with maxillofacial fractures who met the study criteria were included. The study was submitted to the University's ethics committee and was approved by the board and conducted in accordance with the ethical principles of the Declaration of Helsinki.

CBCT images were obtained using a 3D Accuitomo FPD 170 (J. Morita Mfg. Corp., Kyoto, Japan), and CT images were obtained using a 640‐slice scanner (Aquilion ONE, Toshiba Medical Systems, Otawara, Japan).

The patients' gender, age at the time the image was obtained, and month of imaging were noted in the hospital information system. For patients with maxillofacial fractures, the fracture site was categorized as mandible, maxilla (including the alveolar bone, palate, and Lefort I), zygoma, or other (including the orbit, nasal bone, ethmoid, and frontal sinus). The data was recorded as either present or absent. Mandibular fractures were further examined by dividing the mandible into seven regions: the condyle/subcondyle, the ramus, the coronoid process, the angle, the body, the symphysis/parasymphysis (including the canine tooth region of the anterior mandible), and the localized alveolar bone. Additional examinations were performed on patients with mandibular fractures. Total edentulism of the mandible was defined as being edentulous, while the presence of one or more teeth was defined as being dentate. Canine and LTM teeth were also examined. The presence of right, left, and bilateral mandibular canine and premolar teeth was noted. The roots of the LTM teeth were examined in three groups: (1) uncompleted if root development was incomplete; (2) single or fused root for single or fused conical roots; and (3) multiple roots if there were multiple roots separated by furcation. LTM teeth were examined according to Winter's classification [17] based on their angles: horizontal, mesioangular, vertical, and distoangular. The impaction status of the LTM teeth was examined according to the vertical and horizontal position classifications of Pell and Gregory (P&G) [18]. In the P&G classification based on the occlusal plane (vertical position), LTMs were classified as follows: Class A: The highest part of the LTM is at the same level as, or very close to, the occlusal plane of the adjacent second molar tooth. Class B: The highest part of the LTM is below the occlusal plane, but above the enamel‐cementum junction of the adjacent second molar tooth. Class C: The highest part of the LTM is below the cervical line of the adjacent second molar tooth. In the P&G classification relative to the anterior edge of the ramus (horizontal position), Class I: There is sufficient space distal to the lower second molar tooth for the entire crown of the LTM to be positioned in front of the mandibular ramus; Class II: The LTM crown is not sufficiently distal to the mandibular ramus, and the distal part of the LTM crown is embedded in the mandibular ramus. Class III: The LTM is completely or partially embedded in the mandibular ramus. Additionally, it was noted whether the maxillary and mandibular posterior teeth (premolars and molars) on the right and left sides were in occlusion (in contact). Maxillary fractures were also examined in two sections: the anterior maxilla, including the canine, and the posterior maxilla distal to the canine.

### 2.1 Statistical Analysis

The IBM SPSS Statistics (Statistical Package for the Social Sciences, IBM Corp., Armonk, New York, USA) software program, version 27.0, was used for data analysis. Descriptive statistics were performed on the data. Then, Pearson chi‐square analysis was performed to examine the relationships between fracture types (mandible, maxilla, zygoma, and other) and age and gender, as well as the relationships between the season in which the fracture occurred and age and gender. For the chi‐square tests, age was divided into three groups: 20–40 years old, 41–60 years old, and 61 years old and above. Following normality tests, Mann–Whitney *U* and Kruskal–Wallis analyses were performed to examine the relationship between the number of fractures and gender and age groups, depending on the suitability of the data. Binary regression analysis was conducted to investigate the effects of age, gender, the presence of LTMs, the presence of canine teeth, total edentulism, and posterior occlusion on mandibular fractures. Prior to all binary regression analyses, potential multicollinearity among the dental variables included in the models was assessed using variance inflation factor (VIF) and tolerance values, and no significant multicollinearity affecting model stability was detected. Binary regression analysis was also applied to investigate how mandibular angle fractures were affected by the root condition, angle, and vertical and horizontal position of the LTMs according to the Winter and P&G classifications, respectively. Statistical significance was set at *p* < 0.05 in all analyses.

## 3 Results

A total of 466 patients with maxillofacial fractures were included in the study. The mean age of the patients was 37.4 (±14.1) years, with the youngest patient being 20 years old and the oldest patient being 87 years old. Of the patients, 299 (64.2%) were between 20 and 40 years old, 128 (27.5%) were between 41 and 60 years old, and 39 (8.4%) were 61 years old or older. Eighty‐nine patients (19.1%) were female, and 377 patients (80.1%) were male. The gender distribution within age groups is presented in Table 1.

**TABLE 1:** Distribution of cases included in the study by gender and age groups.

Age	Female, *n* (%)	Male, *n* (%)	Total, *n* (%)
20–40	43 (14.4)	256 (85.6)	299 (100)
41–60	36 (28.1)	92 (71.9)	128 (100)
> 60	10 (25.6)	29 (74.4)	39 (100)

No statistically significant differences were observed among age groups with respect to fracture types (mandible, maxilla, zygoma, and others) (*p* > 0.05). Similarly, no difference was found in the number of fracture sites per patient when compared across age groups (*p* = 0.74).

In our study, fractures were most frequently observed in the 20–40 age group during the summer months. However, no statistically significant differences were identified between age groups in terms of the season in which the fracture occurred (*p* = 0.11) (Table 2). Additionally, no statistically significant difference was found between genders with respect to the time of fracture occurrence (*p* = 0.97).

**TABLE 2:** Distribution of cases within age groups according to the time of fracture occurrence.

	Winter, *n* (%)	Spring, *n* (%)	Summer, *n* (%)	Autumn, *n* (%)	*p* *
20–40	55 (58.5)	47 (58.0)	115 (68.4)	82 (66.7)	0.11
41–60	25 (26.6)	27 (33.3)	45 (26.8)	31 (25.2)	
> 60	14 (14.9)	7 (8.6)	8 (4.8)	10 (8.1)	
Total	94 (100)	81 (100)	168 (100)	123 (100)	

* Pearson chi‐square test.

Overall, the majority of patients with maxillofacial fractures were male (80.1%). No statistically significant differences were observed between genders in terms of fracture types or number of fractures (*p* > 0.05 and *p* = 0.29, respectively). However, when gender distribution within age groups was analyzed according to fracture type, mandibular fractures were found to be significantly more frequent among women in the 20–40 age group (*p* = 0.024) (Table 3). No statistically significant differences in gender distribution across age groups were observed for other fracture types (*p* = 0.05).

**TABLE 3:** Distribution of mandible fractures by age groups and gender.

Age groups	Fracture	Female, *n* (%)	Male, *n* (%)	Total, *n* (%)	*p* *
20–40	Present	29 (18.8)	125 (81.2)	154 (100)	0.112
	Absent	14 (9.7)	131 (90.3)	145 (100)	
40–60	Present	18 (28.6)	45 (71.4)	63 (100)	
	Absent	18 (27.7)	47 (72.3)	65 (100)	
> 60	Present	5 (25.0)	15 (75.0)	20 (100)	
	Absent	5 (26.3)	14 (73.7)	19 (100)	

* Pearson chi‐square test.

Of the 466 total patients, 237 (50.9%) had mandibular fractures, 237 (50.9%) had maxillary fractures, 150 (32.2%) had zygomatic fractures, and 218 (46.8%) had fractures of other facial bones, such as the orbit, nasal bone, ethmoid, or frontal sinus. The most common number of fractures was two (*n* = 168, 36.1%), followed by a single fracture (*n* = 149, 32%). The maximum number of fractures in a single patient was seven (*n* = 1, 0.2%) (Table 4).

**TABLE 4:** Number of fracture sites per person.

Number of fracture sites	*n* (%)
1	149 (32)
2	168 (36.1)
3	100 (21.5)
4	35 (7.5)
5	10 (2.1)
6	3 (0.6)
7	1 (0.2)

Table 5 shows the distribution of fractures according to region (mandible, maxilla, zygoma, and other) in all cases, and Table 6 shows the distribution of fractures in cases with mandibular fractures according to mandibular region. Of the maxillary fractures, 232 occurred in the anterior maxilla, 27 occurred in the posterior maxilla, and 22 patients (9.3%) had fractures in both regions.

**TABLE 5:** Distribution of fractures by region in all patients.

Fractured site	*n* (%)
Mandible	184 (39.5)
Maxilla	21 (4.5)
Zygoma	34 (7.3)
Other Bones	8 (1.7)
Mandible + maxilla	4 (0.9)
Mandible + other bones	3 (0.6)
Mandible + maxilla + zygoma	1 (0.2)
Mandible + maxilla + other bones	14 (3)
Maxilla + zygoma	8 (1.7)
Maxilla + other bones	80 (17.2)
Maxilla + zygoma + other bones	78 (16.7)
Mandible + maxilla + zygoma + other bones	31 (6.7)

**TABLE 6:** Distribution of mandible fractures by region.

Fractured site	*n* (%)
Condyle	21 (8.9)
Ramus	8 (3.4)
Coronoid	7 (3.0)
Angle	33 (13.9)
Body	29 (12.2)
Symphysis	22 (9.3)
Alveolar bone	7 (3.0)
Condyle + angle	4 (1.7)
Condyle + body	11 (4.6)
Condyle + symphysis	25 (10.5)
Condyle + alveolar bone	1 (0.4)
Ramus + coronoid	2 (0.8)
Ramus + angle	2 (0.8)
Ramus + body	4 (1.7)
Ramus + symphysis	11 (4.6)
Coronoid + angle	2 (0.8)
Coronoid + body	2 (0.8)
Coronoid + symphysis	1 (0.4)
Angle + body	10 (4.2)
Angle + symphysis	21 (8.9)
Body + symphysis	3 (1.3)
Condyle + ramus + symphysis	1 (0.4)
Condyle + coronoid + body	1 (0.4)
Condyle + coronoid + symphysis	1 (0.4)
Condyle + angle + symphysis	1 (0.4)
Condyle + body + symphysis	1 (0.4)
Ramus + coronoid + angle	1 (0.4)
Ramus + coronoid + body	2 (0.8)
Ramus + coronoid + symphysis	2 (0.8)
Ramus + angle + body	1 (0.4)
Total	237 (100)

Regression analyses were performed separately for each of the seven mandibular regions (condyle/subcondyle, ramus, coronoid, angle, body, symphysis/parasymphysis, and localized alveolar bone). The associations between fracture regions and gender, age, total edentulism, presence of canine, presence of LTM, and posterior occlusion status are presented in Tables 7, 8, 9, 10, 11.

**TABLE 7:** Relationship of mandibular condyle/subcondyle fractures and gender, age, edentulism, presence of canine, presence of LTM and posterior occlusion.

Risk factors	Multivariate		Univariate	
	OR (95% CI)	*p*	OR (95% CI)	*p*
Female gender	0.876 (0.43–1.782)	0.714	0.918 (0.46–1.831)	0.807
Age	1.007 (0.979–1.037)	0.623	1.014 (0.995–1.033)	0.161
Presence of teeth	0.181 (0.011–2.92)	0.228	0.949 (0.244–3.691)	0.940
Presence of canine	2.817 (0.234–33.905)	0.415	1.443 (0.465–4.474)	0.526
Presence of LTM	0.781 (0.365–1.672)	0.525	1.136 (0.628–2.056)	0.673
Having occlusion	2.153 (0.863–5.368)	0.100	2.047 (1.072–3.907)	0.030*
Constant	0.285	0.009		

*Note:* Cox & Snell R Square = 0.029; Nagelkerke R Square = 0.042, Accuracy = 72.2.

* *p* < 0.05.

To further evaluate the association between angular fractures and LTMs, differences in angular fracture frequency were analyzed according to LTM angulation and impaction depth. No statistically significant differences were observed in angular fracture frequency among LTMs with different angulations (horizontal, mesioangular, vertical, and distoangular) on either side, based on Winter's classification (*p* > 0.05). Similarly, no statistically significant differences were identified among P&G classes I, II, and III on the right and left sides (*p* > 0.05).

When P&G depth classifications were evaluated, Class B was found to be 10.7 times more at risk for angular fractures than class A, but only on the right side (*p* = 0.03) (Table 12). The presence or absence of roots was not significantly associated with angular fractures (*p* > 0.05).

The presence of LTMs was also analyzed in relation to condylar fractures, and no statistically significant associations were identified on either the right or left sides (*p* > 0.05).

## 4 Discussion

In our study, 64.2% of cases were between the ages of 20 and 40. This age range is consistent with the peak age range of incidence reported in other studies [11, 12]. Although the incidence was higher in the summer months for our youngest group (aged 20–40), there was no statistically significant difference in the season of fracture occurrence across age groups (*p* = 0.11). Ferreira et al. [1] reported that maxillofacial fractures were significantly more common in children and adolescents during the summer months. However, Stanbouly et al. [19] did not observe a seasonal peak in the geriatric population.

In our study, the majority of individuals affected by maxillofacial fractures were male (80.1%). Many previous studies indicated that males were more prevalent in many sample groups [11, 12, 20, 21, 22]. Ogunbowale et al. [22] reported that men are also more likely than women to have multiple fractures. However, we did not detect a significant difference in the number of fractures based on gender (*p* = 0.29). Only in the 20–40 age group was a statistically significant increase in mandibular fractures observed in women (*p* = 0.024) (Table 3). Contrary to this result, some studies show that the incidence increases with age in women [3, 8, 19, 23]. Ogunbowale et al. [22] identified the 70–89 age range as a peak period for women and the 20–39 age range as a peak period for both genders.

Of the patients, 50.9% had mandibular fractures, 50.9% had maxillary fractures, 32.2% had zygomatic fractures, and 46.8% had fractures of other facial bones. Most patients had two fractures (36.1%), followed by those with a single fracture (32%). The mandible was frequently affected alone, while fractures of the maxilla, zygoma, and other facial bones generally occurred concomitantly with other fractures. Among all patients, 184 (39.5%) had only a mandibular fracture. The next most common were multiple region fractures, such as maxillary and other region fractures (17.2%), and fractures involving the maxilla, other bones, and zygoma (16.7%) (Table 5). A recent review reported that the mandible is the most commonly affected bone in maxillofacial trauma, accounting for an average of 56.6% of cases, and suggested that this higher frequency may be related to its forward and mobile position, which could increase its susceptibility to trauma [12]. Fasola et al. [8] reported that the mandible was affected in 91% of cases of maxillofacial trauma in patients over 60 years of age. However, there are also other studies that have reported the zygomatic bone [20, 22] or the midface bones [3] as the most commonly affected bones in maxillofacial injuries. From a clinical perspective, this distribution highlights the importance of careful mandibular evaluation in trauma patients.

A total of 237 mandibles exhibited fractures in 380 separate regions. The symphysis/parasymphysis region had the most fractures, with 89 cases (19.1%), followed by the mandibular angle with 75 cases (16.1%) and the condyle/subcondyle region with 67 cases (14.4%) (Table 6). Other studies have also reported the symphysis/parasymphysis as the most commonly affected region in mandibular fractures [2, 11, 15]. Kandel et al. [15] found that the most commonly affected regions were the symphysis and parasymphysis (50.1%), followed by the mandibular angle (35.73%) and the condyle (9.74%), which is consistent with our results. However, Morris et al. [10] identified the mandibular angle (27%) as the most commonly affected region, followed by the symphysis (21.3%) and the condyle/subcondyle regions (18.4%). The literature shows that results may vary in age‐specific sample groups. Nagomi et al. [2], for example, identified the symphysis as the most commonly affected region in geriatric patients, followed by the condyle. Fasola et al. [8] found that the mandibular body was the most commonly affected region, followed by the symphysis, and Kannari et al. [23] observed that the condyle and then the symphysis/parasymphysis were the most commonly fractured regions of the mandible in individuals over 60 years old. On the other hand, a study including only children and adolescents up to 18 years of age reported that the condyle and subcondyle were the most commonly affected regions [1]. While our study did not include individuals under 20 years of age, regression analysis revealed that, in adults, the likelihood of fractures in the symphysis and angle was lower with increasing age, while fractures in the body and coronoid regions were more frequently observed in older individuals (Tables 8, 9, 10, 11). Evaluating our findings alongside the literature reveals that symphysis fractures were more frequently observed in adults, while body fractures were more prevalent among geriatric individuals. Clinically, these age‐related variations may help clinicians consider specific mandibular regions during imaging and examination, without implying deterministic patterns.

**TABLE 8:** Relationship of coronoid process of mandible fractures and gender, age, edentulism, presence of canine, presence of LTM and posterior occlusion.

Risk factors	Multivariate		Univariate	
	OR (95% CI)	*p*	OR (95% CI)	*p*
Female gender	1.962 (0.71–5.422)	0.194	0.526 (0.201–1.381)	0.192
Age	1.052 (1.011–1.095)	0.013*	1.032 (1.004–1.06)	0.027*
Presence of teeth	85269812.396 (0–0)	0.999	0.971 (0.118–7.978)	0.978
Presence of canine	0 (0–0)	0.999	1.281 (0.159–10.305)	0.816
Presence of LTM	0.6 (0.195–1.844)	0.372	0.427 (0.173–1.053)	0.064
Having occlusion	0.234 (0.052–1.057)	0.059	1.214 (0.39–3.78)	0.738
Constant	0.021	0.000		

*Note:* Cox & Snell R Square = 0.053; Nagelkerke R Square = 0.117, Accuracy = 90.7.

* *p* < 0.05.

In our study, the two most common fracture combinations were condyle + symphysis/parasymphysis (25, 10.5%) and angle + symphysis/parasymphysis (21, 8.9%) (Table 6). Chen et al. [11] also identified mandibular angle (27.3%) and condyle/subcondyle (21.7%) fractures as the most common dual fracture combinations with symphysis/parasymphysis fractures, but angle + symphysis/parasymphysis fractures were the first. Morris et al. [10] found that angle fractures most commonly accompanied symphysis fractures, followed by condyle/subcondyle fractures. In blows from the chin, the force transmission to the joint area together with the symphysis/parasymphysis has been reported [10, 11, 21]. In fractures combining the angle with the symphysis/parasymphysis, the presence of LTM was observed more frequently in these fracture patterns. Our study revealed that the presence of LTM was associated with a 3.8‐fold higher odds of mandibular angle fracture, indicating a significant association between LTM and mandibular angle fractures under stress conditions in the distal area. Additionally, although the underlying mechanism remains unclear, regression analysis showed that the presence of LTM was also associated with a higher likelihood of symphysis/parasymphysis fractures. Therefore, the relationship between LTM and mandibular angle and symphysis/parasymphysis fractures should be interpreted as an association rather than a causal effect, considering the retrospective design of the study.

Regression analysis revealed a statistically significant relationship between edentulism and fractures of the mandibular body only, with fractures of the mandibular body being more frequently observed in edentulous individuals (Table 10). To our knowledge, no studies have examined the effect of total edentulism on mandibular fractures, making our study the first to report an association between total edentulism and mandibular fracture patterns. Tooth loss has been associated with alveolar bone resorption, which may be related to reduced structural support of the mandibular body. Nogami et al. [2] wrote that, in atrophic mandibles, forces originating from the symphysis have been suggested to be transmitted to the angle and body rather than the symphysis and condyle. Additionally, Fasola et al. [8] found that 60.8% of all mandibular fractures in their study of facial fractures in geriatric individuals occurred in the body. Age‐related molecular changes in bone have been reported to reduce bone resistance to trauma [20]; however, these changes may influence different mandibular regions to varying degrees. Mundinger et al. [3] found that geriatric patients had a significantly lower incidence of both mandible fractures and mandibular body, angle, condyle (excluding the subcondyle), parasymphysis, and symphysis fractures of the mandible compared to non‐geriatric patients. In our study, we did not detect a significant difference in mandibular fractures in the group aged 60 and over. However, age, total edentulism, absence of posterior occlusion, absence of canine, and absence of LTM teeth were identified as variables significantly associated with fractures of the mandibular body (Table 10). Taken together, these findings suggest that fractures of the mandibular body are more frequently observed in older individuals compared with younger individuals, within the limitations of this retrospective analysis.

The effects of canine teeth and LTMs were examined separately. The presence of canine teeth was found to be associated with mandibular body fractures, with a lower likelihood of body fractures observed when canines were present. Based on the possibility that canine teeth may create a wedge‐like effect in the parasymphyseal region due to their anatomical characteristics including large and long roots, we aimed to investigate the relationship between the presence of canines and the fracture site. To our knowledge, this is the first study to examine the relationship between mandibular fractures and canine teeth. Although fractures of the parasymphysis were more frequently observed than body fractures in individuals with canines, regression analysis demonstrated a statistically significant association only between the presence of canines and a reduced likelihood of mandibular body fractures. Future studies that take canine teeth into account in mandibular fractures could help clarify their potential influence on fracture patterns.

A significant relationship was found between the presence of the LTM and fractures of the mandibular angle, body, and symphysis/parasymphysis (*p* < 0.05). The presence of an LTM was associated with a 3.8‐fold higher odds of mandibular angle fractures (Table 9). Another significant finding was that the likelihood of mandibular body fractures was 32% higher in the absence of LTM. These findings appear to be complementary, suggesting that fracture patterns may vary according to the presence or absence of LTM. In this context, the presence of LTM was associated with a higher frequency of fractures in the mandibular angle region, whereas in the absence of LTM, fractures were more frequently observed in more anterior regions, such as the mandibular body, following traumatic exposure. Nogami et al. [9] and Kandel et al. [15] found that the incidence of mandibular angle fractures was significantly higher in the presence of LTM. Three meta‐analysis studies in the literature have reported that the presence of LTM is associated with an increased likelihood of mandibular angle fractures, with reported odds ratios ranging from 1.9 to 3.8 [13, 14, 24]. The third region found to be statistically significantly associated with the presence of LTM in our study was the symphysis/parasymphysis region (*p* = 0.01). The observed 2.15‐fold higher odds (1.19–3.871) of symphysis/parasymphysis fractures in the presence of LTM suggests that complex patterns of force transmission may be involved (Table 11). These associations may help clinicians recognize potential fracture distributions in the context of dental status, but they should not be used as stand‐alone predictors.

**TABLE 9:** Relationship of mandibular angle fractures and gender, age, edentulism, presence of canine, presence of LTM and posterior occlusion.

Risk factors	Multivariate		Univariate	
	OR (95% CI)	*p*	OR (95% CI)	*p*
Female gender	0.862 (0.417–1.779)	0.688	1.335 (0.673–2.649)	0.408
Age	0.969 (0.937–1.001)	0.061	0.957 (0.934–0.979)	< 0.001*
Presence of teeth	3.324 (0–0)	1.000	802388855.031 (0–0)	0.999
Presence of canine	755048660.727 (0–0)	0.999	818653472.74 (0–0)	0.998
Presence of LTM	2.355 (1.071–5.179)	0.033	3.798 (1.902–7.585)	< 0.001*
Having occlusion	1.546 (0.538–4.445)	0.419	0.377 (0.173–0.822)	0.014*
Constant	0.000	0.999		

*Note:* Cox & Snell R Square = 0.105; Nagelkerke R Square = 0.147, Accuracy = 68.4.

* *p* < 0.05.

**TABLE 10:** Relationship of mandibular body fractures with gender, age, edentulism, presence of canines, presence of LTM and posterior occlusion.

Risk factors	Multivariate		Univariate	
	OR (95% CI)	*p*	OR (95% CI)	*p*
Female gender	0.679 (0.312–1.476)	0.328	1.142 (0.564–2.312)	0.713
Age	0.998 (0.968–1.029)	0.898	1.033 (1.013–1.053)	0.001*
Presence of teeth	0 (0–0)	0.999	0.071 (0.015–0.341)	0.001*
Presence of canine	1021754128.213 (0–0)	0.999	0.182 (0.058–0.566)	0.003*
Presence of LTM	0.45 (0.213–0.949)	0.036*	0.320 (0.176–0.58)	< 0.001*
Having occlusion	1.76 (0.687–4.504)	0.239	0.322 (0.168–0.615)	0.001*
Constant	3.386	0.302		

*Note:* Cox & Snell R Square = 0.109; Nagelkerke R Square = 0.159, Accuracy = 75.9.

* *p* < 0.05.

**TABLE 11:** Relationship of mandibular symphysis/parasymphysis fractures with gender, age, edentulism, presence of canines, presence of LTM and posterior occlusion.

Risk factors	Multivariate		Univariate	
	OR (95% CI)	*p*	OR (95% CI)	*p*
Female gender	0.851 (0.433–1.673)	0.639	1.312 (0.685–2.514)	0.413
Age	0.993 (0.965–1.021)	0.607	0.974 (0.954–0.993)	0.009*
Presence of teeth	2579541744.44 (0–0)	0.999	1049469958.419 (0–0)	0.999
Presence of canine	0.175 (0.014–2.216)	0.178	3.838 (0.839–17.566)	0.083
Presence of LTM	1.452 (0.718–2.937)	0.299	2.147 (1.19–3.871)	0.011*
Having occlusion	0.693 (0.27–1.78)	0.446	0.421 (0.207–0.854)	0.017*
Constant	0.000	0.999		

*Note:* Cox & Snell R Square = 0.070; Nagelkerke R Square = 0.096, Accuracy = 62.9.

* *p* < 0.05.

**TABLE 12:** The relationship between LTM status and mandibular angle fracture in the right mandible.

Risk factors	OR (95% CI)	*p*
LTM root (uncompleted)		
Single/fused	1.526 (0.107–21.781)	0.755
Mutiple	1.459 (0.107–19.859)	0.777
Pell‐Gregory (A)		
B	10.672 (1.193–95.493)	0.034*
C	5.494 (0.84–35.938)	0.075
Pell‐Gregory (I)		
II	0.115 (0.013–1.01)	0.051
III	1898387611.899 (0–0)	1.000
Winter (vertical)		
Mesioangular	1.538 (0.477–4.955)	0.471
Distoangular	2.368 (0.317–17.709)	0.401
Horizontal	8.753 (0.743–103.065)	0.085
Constant	0.106 (0–0)	0.095

*Note:* Cox & Snell R Square = 0.076; Nagelkerke R Square = 0.125, Accuracy = 82.4.

* Multivariate logistic regression (*p* < 0.05).

The difference in angle and impaction degree of LTMs in terms of angular fractures was only detected on the right side between P&G classes A, B, and C (see Table 12). Class B was associated with a 10.7‐fold higher odds of angular fractures compared with Class A (*p* = 0.03). Our finding that the angulation of the LTM was not significantly associated with mandibular angle fractures is consistent with previous studies [9, 13]. However, previous studies have reported various findings regarding LTM conditions according to the P&G classifications. Nogami et al. [9] found that fully erupted (class A) and partially bone‐retained (class B) LTMs increased the risk of angular fractures compared to fully bone‐retained LTMs (class C). In contrast, Armond et al. [13] reported that Class A and Class I LTMs reduce the risk of angular fractures while Class B and Class II AUMs were associated with a 1.4 and 1.7 fold higher odds, respectively. Meta‐analyses conducted by Giovacchini et al. [14] and Kandel et al. [15] support the association between the presence of LTM and mandibular angle fractures, reporting that the likelihood of angular fractures increases with greater impaction depth in both the vertical (A–C) and horizontal (I–III) directions. Although deeper LTM impaction may be hypothesized to be associated with reduced bone continuity, the variability among reported findings suggests that factors other than the type of LTM impaction may play a more prominent role in the occurrence of angular fractures.

In addition to angular fractures, the literature has also focused on the relationship between LTMs and condylar fractures. Some studies have shown that the presence of LTM is associated with a higher likelihood of mandibular angular fractures, while the absence of LTM is associated with a higher likelihood of condylar fractures [9, 21, 24]. In the absence of LTM, it has been suggested that the mandibular angle region may exhibit greater structural continuity, allowing forces originating from the symphysis to be transmitted through the angle to the ramus and condyle, potentially resulting in condylar fractures; however, in the presence of LTM, force transmission may be cut off due to the occurrence of angle fractures [9]. Despite that, Armond et al. [13] reported in their meta‐analysis that the presence of LTM was associated with a lower likelihood of condylar fracture. In our study, we did not detect an effect of the LTM's presence on condylar fractures (*p* > 0.05) (Table 7). Cases with isolated condyle/subcondyle fractures accounted for 31.3% (21 out of 67) of condyle/subcondyle fracture cases. Similar to Antic et al. [21], this finding suggests that direct impact to the condyle may be relatively uncommon, and that condyle/subcondyle fractures are frequently observed in association with trauma to other mandibular regions, such as the symphysis. Within this multifactorial process, the magnitude and direction of traumatic forces may have a greater influence on fracture patterns than the presence of LTM alone.

Among the 237 patients with mandibular fractures, 185 (78.1%) exhibited posterior occlusion, while 52 (21.9%) did not. One of the most notable findings of our study was that posterior mandibular occlusion was associated with a lower likelihood of fractures in the mandibular angle, body, and symphysis/parasymphysis, whereas a higher likelihood was observed for condyle/subcondyle fractures (*p* < 0.05) (Tables 7, 9, 10, 11). The presence of posterior occlusion was associated with a 2.05‐fold higher odds (1.072–3.907) of condyle/subcondyle fractures. Apart from the current study, only one other study in the literature has evaluated posterior tooth occlusion in relation to mandibular fractures [2]. Nogami et al. [2] reported that condylar fractures, including those concomitant with symphysis fractures, were less frequently observed in cases with posterior occlusion compared to those without, and suggested that the condylar region was protected by the distribution of forces, especially those originating from the symphysis region, due to contact between the posterior lower and upper teeth. Posterior occlusion may be associated with a lower likelihood of fractures in the mandibular angle, body, and symphysis/parasymphysis, potentially through force transmission and mechanical support to the mandible in a direction opposing the applied force, particularly during downward–upward blows. However, under horizontal anteriorly directed forces, loss of intercuspation may occur. Additionally, in the absence of occlusion, the mandible may rotate upward following a blow; however, occlusal contact may limit this movement through a stopper‐like effect. As a result, forces may be concentrated in the condyle/subcondyle region, which could be associated with a higher frequency of condylar fractures in cases with posterior occlusion (Figure 1). These biomechanical considerations are proposed to contextualize the observed associations and should not be interpreted as causal mechanisms.

**FIGURE 1:**
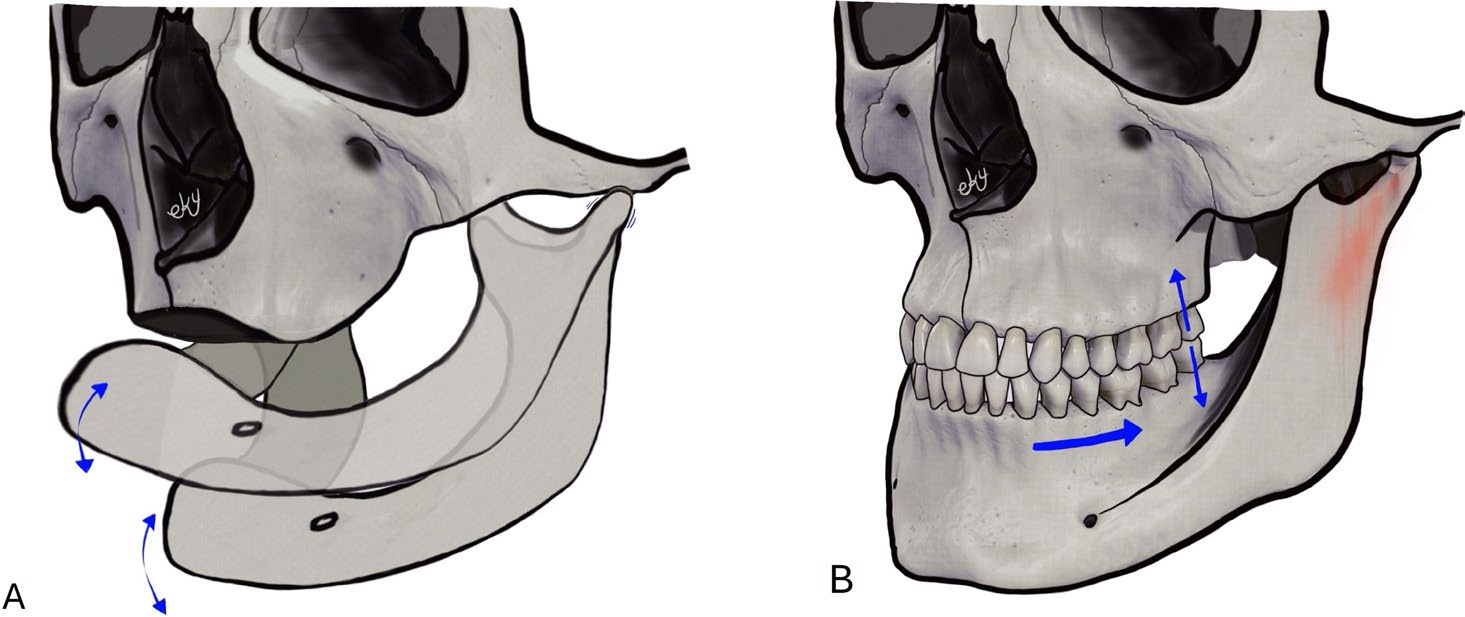
The potential impact of an upward blow to the chin on the condylar region is illustrated in mandibles with and without posterior occlusion. (A) demonstrates the free swing of the mandible in the absence of posterior occlusion, while (B) highlights the stress concentration in the condylar region due to the stopper effect of occlusion.

Because the study was retrospective access to detailed information regarding the circumstances of each case was limited. For instance, data on the mechanism of injury, such as falls, traffic accidents, or physical assault, were not available. Such information could contribute to a more comprehensive understanding of how different trauma mechanisms may be associated with fracture patterns. Similarly, information regarding whether injuries occurred at home, at work, or during sports activities, and how these factors relate to age and gender, could provide valuable sociological perspectives. Past studies have reported that road traffic accidents represent a major etiological factor in maxillofacial fractures [8, 15, 20]. In younger age groups, it is possible to specifically mention motor vehicle accidents [1]. Conversely, falls and home accidents have been reported more frequently in geriatric populations [3, 8, 19, 20]. Additionally, physical assault has been identified as another important etiological factor in the general population [15, 21]. Future studies incorporating such variables may help to better characterize these associations and inform preventive strategies.

This study examined maxillofacial fractures in terms of their anatomical distribution, the combination of bones affected, age, and gender. In addition, mandibular fractures were examined in relation to several dental conditions, including lower third molars, canines, total edentulism, and posterior occlusion, thereby providing comprehensive data on the associations between dental status and fracture patterns.

Maxillofacial fractures were observed more frequently in males, with the maxilla and mandible being the most commonly affected bones. Among cases involving a single bone, the mandible was most frequently affected. Mandibular fractures were most prevalent in the symphysis/parasymphysis region (19.1%) and the mandibular angle region (16.1%). Condylar/subcondylar fractures occurred more frequently concurrently with fractures in other mandibular regions, such as the symphysis/parasymphysis. Increasing age was associated with a higher frequency of coronoid and mandibular body fractures, while fractures of the mandibular angle and symphysis/parasymphysis were observed less often. Total edentulism and absence of the canine tooth were associated with a higher prevalence of mandibular body fractures. Posterior occlusion was associated with lower frequencies of fractures in the mandibular body, angle, and symphysis/parasymphysis regions, while a higher frequency of condylar fractures was observed in individuals with posterior occlusion. The presence of a lower third molar was associated with a higher prevalence of mandibular angle fractures. Furthermore, Pell and Gregory Class B lower third molars were associated with a markedly higher frequency of angle fractures, even when present unilaterally.

Within the limitations of this retrospective observational study, these findings provide clinically relevant information for the evaluation of patients presenting with maxillofacial trauma. Considering these associations, clinicians may find it helpful to contextualize fracture patterns during diagnostic evaluation, taking into account dental characteristics alongside clinical and radiological findings. Awareness of the observed associations between the condylar/subcondylar fractures and posterior occlusion; mandibular body fractures and edentulism and advanced age; and mandibular angle and symphysis/parasymphysis fractures and the presence of LTM teeth may facilitate a more efficient clinical assessment.

## Author Contributions

Canan Uzun: Study design, data interpretation, statistical analysis, manuscript writing, and final approval of the manuscript. Ceyhun Uzun: Study design, data collection, data interpretation, and final approval of the manuscript. Savas Ozarslanturk: Data collection, data interpretation, and final approval of the manuscript. Emrah Kagan Yasar: Data collection, illustration drawing, and final approval of the manuscript.

## Funding

The authors have nothing to report.

## Conflicts of Interest

The authors declare no conflicts of interest.

## Data Availability

The data that support the findings of this study are available on request from the corresponding author. The data are not publicly available due to privacy or ethical restrictions.
